# Neural representations underlying mental imagery as unveiled by representation similarity analysis

**DOI:** 10.1007/s00429-021-02266-z

**Published:** 2021-04-05

**Authors:** Maddalena Boccia, Valentina Sulpizio, Federica Bencivenga, Cecilia Guariglia, Gaspare Galati

**Affiliations:** 1 Department of Psychology, “Sapienza” University of Rome, Via dei Marsi, 78, 00185 Rome, Italy; 2 Cognitive and Motor Rehabilitation and Neuroimaging Unit, Santa Lucia Foundation (IRCCS Fondazione Santa Lucia), Rome, Italy; 3 PhD Program in Behavioral Neuroscience, Sapienza University of Rome, Rome, Italy

**Keywords:** Visual mental images, Perception, fMRI, Multivariate pattern analysis, Representation similarity analysis

## Abstract

It is commonly acknowledged that visual imagery and perception rely on the same content-dependent brain areas in the high-level visual cortex (HVC). However, the way in which our brain processes and organizes previous acquired knowledge to allow the generation of mental images is still a matter of debate. Here, we performed a representation similarity analysis of three previous fMRI experiments conducted in our laboratory to characterize the neural representation underlying imagery and perception of objects, buildings and faces and to disclose possible dissimilarities in the neural structure of such representations. To this aim, we built representational dissimilarity matrices (RDMs) by computing multivariate distances between the activity patterns associated with each pair of stimuli in the content-dependent areas of the HVC and HC. We found that spatial information is widely coded in the HVC during perception (i.e. RSC, PPA and OPA) and imagery (OPA and PPA). Also, visual information seems to be coded in both preferred and non-preferred regions of the HVC, supporting a distributed view of encoding. Overall, the present results shed light upon the spatial coding of imagined and perceived exemplars in the HVC.

**Supplementary Information:**

The online version contains supplementary material available at 10.1007/s00429-021-02266-z.

## Introduction

Mental imagery corresponds to the human ability to access perceptual information from memory to create a complex and sophisticated mental experience of objects, people, or places (Farah 1989; Kosslyn 1980). The study of the role of the early visual cortex (in particular V1) has dominated brain imaging work on mental imagery for the last 25 year, yielding sometimes contradictory findings (for reviews, see Kosslyn and Thompson 2003; Pearson 2019). Conversely, it seems well established that mental imagery critically depends on the content-dependent brain areas in the occipito-temporal high-level visual cortex (HVC), which activation depends on the object category (i.e., faces, places, objects, body parts; O’Craven and Kanwisher 2000), and on the hippocampus (HC; Boccia et al. 2015). Indeed, mental imagery and visual perception have been found to rely on the same content-dependent brain areas in the HVC: imagining a face leads to the activation of the fusiform face area (FFA), which selectively responds during face perception; similarly, imagining a scene leads to the activation of the parahippocampal place area (PPA), which selectively responds during scene perception (Ishai et al. 2000; O’Craven and Kanwisher 2000). The HC has been found to be involved in mental imagery in general (Boccia et al. 2015), with different connectivity patterns depending on the content of the mental image (Boccia et al. 2017, 2019). Interestingly, Reddy et al. (2010) found that category information could be reliably decoded from the ventral temporal cortex, but not from the early retinotopic brain areas.

Studies using multivariate pattern analysis—namely, the information-based approach aimed at identifying a perceptual representation or cognitive state on the basis of multi-voxel regional fMRI signals (Kriegeskorte and Bandettini 2007a, b)—undermine a strong “modular view” of stimuli representation in the HVC. Since the seminal paper by Haxby and colleagues (Haxby et al. 2001), in which the authors used correlations between response patterns as an index of similarity, it has become clear that the representations of faces and objects in the HVC may be widely distributed. Indeed, these authors found that the category of the presented stimulus (e.g., a face) could be identified from the distributed pattern of activity in the HVC even after excluding the area that maximally responded to that category (e.g., FFA) from the analysis, and even when limiting the analysis to regions maximally responding to another category (e.g., PPA). Consistent with these results, O’Toole and colleagues (O’Toole et al. 2005) found that both preferred and non-preferred regions can provide good, almost comparable, information for object classification. Also, shared attributes of object structure were reflected in the similarity patterns of brain responses to these attributes. This evidence points towards a “distributed view” of object representation within the HVC, as previously proposed by Ishai and colleagues (Ishai et al. 1999). More specifically, these results tie well with the object-form topography hypothesis, which assumes that the neural encoding of object attributes is distributed, since HVC holds a continuous representation of objects in terms of their attributes or features (Haxby et al. 2001). In this vein, different perceptual categories share the neural space when they share common attributes (O’Toole et al. 2005).

Which are the implications of the modular and the distributed views for mental imagery? In the past 5 years, we performed a number of functional magnetic resonance imaging (fMRI) experiments aimed at testing the structure of spatial and perceptual information of mental images. In a first preliminary experiment (Boccia et al. 2015), we found that topological mental images—namely, images in which it is possible to navigate (Guariglia and Pizzamiglio 2006, 2007)—activate the same scene-selective brain regions required for perception of landmarks in the HVC, i.e., the PPA and the retrosplenial complex (RSC); whereas, non-topological images—namely, images in which it is not possible to navigate (Guariglia and Pizzamiglio 2006, 2007)—activate a different set of brain areas. This result ties well with the idea that a certain degree of specificity does exist for the content of the mental imagery and with the modular view. However, spatial coding of both topological and non-topological images, assessed using multi-voxel pattern classification (Kriegeskorte and Bandettini 2007a, b), was widely distributed in the brain (Boccia et al. 2015). In a second fMRI experiment (Boccia et al. 2017), using multi-voxel pattern classification (Kriegeskorte and Bandettini 2007a, b), we found that item-specific information from perceived landmarks was re-instantiated during mental imagery of the same landmarks (and vice versa) in scene-selective regions (i.e., PPA and RSC) as well as in the HC. In a third fMRI experiment (Boccia et al. 2019), we tested the regional specificity of such a representation, and found that, besides generalizing across imagery and perception, item-specific information about faces and landmarks is widely coded in the HVC, in both preferred and non-preferred content-dependent regions: indeed, both face-selective regions, i.e., FFA and the occipital face area (OFA), and scene-selective regions, i.e., PPA, RSC, and the occipital place area (OPA), significantly decoded items from both the preferred and the non-preferred perceptual category. This result is in line with the idea that content-dependent regions of the HVC share the representations of the perceptual category (i.e., place or faces) about both preferred and non-preferred categories and with the distributed view (Haxby et al. 2001; O’Toole et al. 2005). However, in consistence with the modular view, fine-grained information about exemplars (e.g., landmark or face identity), assessed using representation similarity analysis (RSA; Boccia et al. 2019), was clearly structured in macro-blocks along the category and task boundaries in the content-dependent regions of the HVC and mirrored their perceptual preference. Also, different regional mechanisms and inter-regional functional couplings subtend imagery and perception of different perceptual categories, in agreement with previous neuropsychological findings (Boccia et al. 2018; Committeri et al. 2015).

Here, we re-analyzed data from these previous fMRI experiments to unveil how (i.e., in a distributed or a modular fashion) the fine-grained spatial and visual information about perceived and imagined exemplars are coded in service of mental imagery in the HVC, and possible differences between imagery and perception. To this aim, we performed a RSA on previous data collected during imagery and perception of topological (i.e. familiar buildings/landmarks) and non-topological (i.e., cities on the map of Italy, hours on the clock and familiar faces) categories of exemplars. As opposed to our previous study using RSA (Boccia et al. 2019), here we took advantage of the cross-validated Mahalanobis estimator (Diedrichsen et al. 2016; Walther et al. 2016) to compute distances that follow a t-distribution. Estimating neural dissimilarities with a meaningful zero point allowed us to perform direct statistical comparisons across domains (namely, imagery and perception) and categories. Furthermore, beyond testing the neural signature of these categories, and relative exemplars in the HVC and the HC, at difference with the previous study we also focused on the spatial information they eventually conveyed. Based on previous literature, we predicted that fine-grained spatial information about topological mental images is coded within the scene-selective regions of the HVC and that spatial similarities between imagined and perceived buildings do not differ (Boccia et al. 2017). We also predicted that visual similarities between perceived items partially differ from imagined ones (Lee et al. 2012; Boccia et al. 2019).

## Materials and methods

### Participants

We re-analyzed data from 48 healthy right-handed individuals who took part in our previous studies, as it follows: 15 individuals (mean age: 24.67 and SD: 2.16; seven women) took part in the Experiment 1 (Boccia et al. 2015); 16 individuals (mean age: 26.31 and SD: 2.80; 3 women) took part in the Experiment 2 (Boccia et al. 2017); and 19 individuals (mean age: 24.95 and SD: 1.84; 8 women) took part in the Experiment 3 (Boccia et al. 2019). Two individuals took part in both experiments 1 and 2.

All participants were students at the Sapienza University of Rome and, thus, very familiar with the university campus (Boccia et al. 2015, 2017, 2019). Campus knowledge was assessed with a preliminary questionnaire in which participants were asked to locate 15 campus buildings on an outline map. Participants of the Experiment 3 were also familiar with famous faces selected for the study as supported by performances on a preliminary questionnaire in which participants were asked to link 12 famous people's faces and names.

All participants gave their written informed consent to participate in the studies. The studies were designed in accordance with the principles of the Declaration of Helsinki and were approved by the ethical committee of Fondazione Santa Lucia, Rome.

### Stimuli and procedures

All experiments were developed as fMRI event-related paradigms using a continuous carry-over design (Boccia et al. 2015, 2017, 2019). Thus, during fMRI scans, stimuli were presented in an unbroken sequential manner in serially balanced sequences in which each stimulus preceded and followed every other stimulus (Aguirre 2007; Nonyane and Theobald 2007). Stimuli of each experiment, along with specific experimental design, are described below. For each experiment, design is summarized in Table 1.

**Table 1:** For each experiment, the number of participants, the number of experimental trials and conditions are listed, along with the number of stimuli presented using carry-over sequence

	Experiment 1	Experiment 2	Experiment 3
Participants	15	16	19
Number of trials*	600	288	600
Imagined cities	8^@^		
Imagined clock	8^@^		
Imagined buildings	8^@^	8^@^	6^§^
Imagined faces			6^§^
Perceived buildings		8^@^	6^§^
Perceived faces			6^§^

*Overall experimental trials in each experiment

^@^Individuals were explicitly asked to imagine the spatial position of items

^§^Individuals were not explicitly asked to imagine the spatial position of items

#### Experiment 1

In each trial, individuals were asked to imagine as vividly as possible a building within the university campus, or a city on the map of Italy, or an hour on the clock, and its relative spatial position. For each category, we chose eight target items (Campus: Department of Literature, Department of Mathematics, Department of Chemistry, Orthopedics Clinic, Institute of Hygiene, Chapel, Department of Political Science, Department of Law; Italian cities: Bolzano, Trieste, Lecce, Foggia, Salerno, Frosinone, Alessandria, Cuneo; times on the clock face: 01:00, 02:00, 04:00, 05:00, 07:00, 08:00, 10:00, 11:00). For all the three categories, items were placed so that there were two items in each of four spatial quadrants (i.e., north-east, south-east, south-west, north-west; Supplementary Figure S1A). The sub-division in quadrants was used to test whether the neural representations reflect information about the spatial location of the imagined item (see more details below) and possible differences between categories.

Participants were given written instructions about the item to be imagined and asked to imagine the corresponding building/city/hour and its spatial position as vividly as possible (Supplementary Figure S1B). They were also advised that questions could appear in a random order (question trials). Question trials were introduced to ensure that subjects executed the imagination task. Nine questions appeared randomly and concerned the spatial position of the latest item with respect to another one presented in the question trial. For example, “Is it on the right of 10:00?” or “Is it further north than Lecce?” or “Is it on the right of the Department of Literature?”. Participants had to respond using either of two buttons on the fMRI-compatible keypad. They were scanned during five fMRI scans consisting of 277 fMR volumes (further details about image acquisition are provided below). Each category item was presented five times in each fMRI scan. Thus, each scan consisted of 120 experimental trials, plus 5 null trials and 9 question trials. The question trials lasted 4 s; whereas, all the remaining trials lasted 2 s and were followed by a fixation point of the same duration.

#### Experiment 2

In the second experiment, individuals were scanned while viewing or imagining buildings within the university campus. The same set of stimuli from Experiment 1 was used (i.e., written labels), along with the corresponding photos (Supplementary Figure S2A). Similar to the Experiment 1, participants were asked to pay attention to each stimulus and imagine or watch the building and its relative spatial position (Supplementary Figure S2B). They were advised that questions concerning the spatial position of the latest item could appear randomly: as for the Experiment 1, questions concerned the spatial position of the latest item with respect to another one presented in the question trial. Participants answered using one of the two buttons on the fMRI-compatible keypad. Each item was presented six times in each of the three fMRI scans. Each scan (238 fMR volumes) consisted of 48 perceptual and 48 imagery experimental trials, plus 12 null trials and 6 question trials. Similar to the Experiment 1, trials lasted 2 s and were followed by a fixation point of the same duration; whereas, the question trials lasted 4 s.

#### Experiment 3

In the third experiment, individuals were scanned while viewing or imagining buildings in the university campus or familiar faces (Supplementary Figure S3A). A subset of stimuli from Experiments 1 and 2 (i.e., Department of Literature, Orthopedics Clinic, Institute of Hygiene, Chapel, Department of Mathematics, Department of Law) was used, along with new stimuli, both pictures and written labels, about famous people. Participants were provided with the photo of the familiar landmark/famous face (during perceptual trials) or its name (during imagery trials) and were asked to watch the stimulus during perception or imagine the indicated stimulus during imagery task (Supplementary Figure S3B). Again, questions could appear randomly and concerned perceptual details of the last imagined/perceived item. For example, “Are there trees in front of it?” or “Has she got brown hair?”; participants responded using one of the two buttons on the fMRI-compatible keypad. Five fMRI scans consisting of 277 fMR volumes were acquired. Each item was presented 5 times in each fMRI scan; each scan consisted of 60 perceptual (half were landmarks) and 60 imagery (half were landmarks) trials, plus 5 null trials and 9 question trials. With the exception of the question trials, which lasted 4 s, trials lasted 2 s and were followed by a fixation point of the same duration.

### Image acquisition

A Siemens Allegra scanner (Siemens Medical Systems, Erlangen, Germany), operating at 3 T and equipped for echo-planar imaging was used to acquire functional magnetic resonance images. Head movements were minimized with mild restraint and cushioning. Stimuli were generated by a control computer located outside the MR room, running in-house software implemented in MATLAB. An LCD video projector projected stimuli to a back-projection screen mounted inside the MR tube and visible through a mirror mounted inside the head coil. Presentation timing was controlled and triggered by the acquisition of fMRI images. Functional MRI images were acquired for the entire cortex using blood-oxygen-level-dependent (BOLD) contrast imaging (30 slices, in-plane resolution = 3 × 3 mm, slice spacing 4.5 mm, repetition time [TR] = 2 s, echo time [TE] = 30 ms, flip angle = 70 deg). We also acquired a three-dimensional high-resolution T1-weighted structural image for each subject (Siemens MPRAGE, 176 slices, in-plane resolution = 0.5 × 0.5 mm, slice thickness = 1 mm, TR = 2 s, TE = 4.38 ms, flip angle = 8 deg).

### Regions of interest (ROIs)

RSA was conducted on independently defined, theoretically motivated, regions of interest (ROIs) within the HVC, i.e., scene-selective regions (parahippocampal place area—PPA, occipital place area—OPA, and retrosplenial complex—RSC), face-selective regions (fusiform face area—FFA, and occipital face area—OFA), and in the hippocampus (HC).

**Fig. 1:**
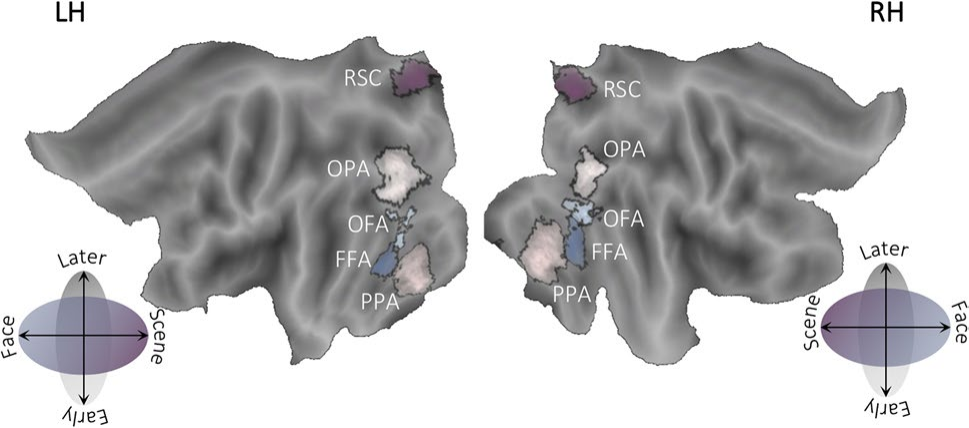
Regions of interest in the HVC. Scene- and face-responsive ROIs correspond to the regions responding more strongly to places/scenes than to faces, (OPA, PPA and RSC), in light violet-to dark violet, and those responding more strongly to faces than to places/scenes (OFA and FFA), in light blue-to-dark blue

Both scene- and face-selective regions were identified by analyzing data from independent ‘‘localizer’’ scans performed with participants of Experiment 3 (for details about sequences and procedure, see Boccia et al. 2019) in which place/scene and face blocks were modeled as box-car functions, convolved with a canonical hemodynamic response function. Scene-selective areas were created as the regions responding stronger to pictures of scenes/places than to pictures of faces in the parahippocampal cortex (i.e., PPA), in the retrosplenial/parieto-occipital sulcus (i.e., RSC), at the junction with the anterior calcarine sulcus, and in the lateral occipital cortex (i.e., OPA). Face-selective areas were created as the regions responding stronger to pictures of faces than to pictures of scenes/places in the fusiform gyrus (i.e., FFA) and inferior occipital cortex (i.e., OFA). These ROIs were created on each individual's brain surface by selecting single activation peaks from the statistical maps and their neighborhood through a watershed segmentation algorithm as applied to surface meshes (Mangan and Whitaker 1999). We then averaged individual ROIs creating probabilistically defined ROIs. We thresholded the probabilistic ROIs to keep only the nodes that were present in at least the 20% of the subjects. In other words, we excluded the nodes that were shared across less than 20% of the subjects, thus selecting the nodes which are representative of the most common location of such regions. The HC, instead, was defined on an anatomical basis in each participant based on the automatic segmentation provided by FreeSurfer (Van Leemput et al. 2009). Figure 1 shows the anatomical location of the probabilistically defined scene- and face-selective regions overlaid onto the flattened Conte69 atlas surface (Van Essen et al. 2012). ROIs derived from our localizer analyses are comparable to ROIs derived from a meta-analysis (see Supplementary Figure S4).

### Image analysis

#### General linear model

After standard preprocessing and resampling onto individually reconstructed cortical surfaces (see for example Boccia et al. 2019), we used a general linear model (GLM) on unsmoothed time series, in which trials related to each exemplar of each category (i.e., building, city, hour, or face) within each domain (i.e., imagery or perception) were modeled by separate regressors, to estimate the magnitude of the response at each voxel/node for each exemplar and domain separately. As nuisance regressor, we included the framewise displacement (FD), a subject-specific time-series index of the overall estimate of movement over time. FD is computed as the sum of the absolute temporal derivatives of the six head-movement-related parameters (three for translations and three for rotations).

#### Representational dissimilarity matrices

For all the experiments, multi-voxel patterns of activity for each exemplar were extracted in scene-selective regions (parahippocampal place area—PPA, occipital place area—OPA, and retrosplenial complex—RSC) and the hippocampus (HC), as vectors of scaled parameter estimates from the GLM regressors corresponding to each exemplar. Multi-voxel patterns of activity were extracted also in face-selective regions (fusiform face area—FFA, and occipital face area—OFA) for the Experiment 3.

For each region, we built a representational dissimilarity matrix (RDM) by computing cross-validated Mahalanobis (crossnobis) distances (Diedrichsen et al. 2016; Walther et al. 2016) between the activity patterns associated with each pair of stimuli, as an index of dissimilarity of neural representations. This measure includes a multivariate noise normalization (Arbuckle et al. 2018; Walther et al. 2016) and, differently from other distance measures (e.g. Euclidean distance), it provides a meaningful zero point by computing dissimilarities through a leave-one-out cross-validation scheme. As a first step, separately for each run, we suppressed correlated noise across voxels by applying the multivariate noise normalization. We then computed the dissimilarity (d_i,j_) between the activation patterns (u) of a pair of stimuli (i,j) using a leave-one-out cross validation scheme as follows (Beukema et al. 2019):

$${d}_{i,j}= {\sum }_{l,m; l\ne m}^{M} \frac{({u}_{i}^{m}-{u}_{j}^{m}{)}^{T}({u}_{i}^{l}-{u}_{j}^{l}) }{M(M-1)}$$

where *M* represents the independent partitions (cross-validation folds), and *T* the number of time points. Distances were computed in each pair of runs (*l*,*m*) and then averaged across each possible combination of runs. The cross-validation ensures that the resulting distances will not be biased by run-specific noise, since noise is assumed to be orthogonal across runs. If the true distance between a pair of stimuli is zero (i.e., maximum similarity), the corresponding value of the crossnobis distance will be zero (i.e., in the leading diagonal of an RDM); if two stimuli consistently induce different patterns of activity, the crossnobis distance will be positive. Cross-validation also allows negative crossnobis distances, if the pattern of activity of each stimulus is not consistent and, consequently, a given area is not reliably able to encode differences between them. The meaningful zero point allows testing cross-validated Mahalanobis distances against zero to assess whether an area significantly discriminates between a pair of stimuli (i.e., the average distance will be significantly higher than zero) or not (Beukema et al. 2019; Diedrichsen et al. 2016; Diedrichsen and Kriegeskorte 2017; Walther et al. 2016). Furthermore, it is also possible to compare distances between two or more pair of stimuli by means of two-sample or paired *t*-tests, to assess whether a distance is higher than others (Diedrichsen et al. 2016; Walther et al. 2016; Yokoi et al. 2018).

We then averaged the RDM elements, within subjects, depending on whether they were computed on pairs of stimuli belonging to the same or different domains (imagery, perception), to the same or different categories (buildings, cities, hours, faces), and to the same or different spatial quadrants (north-east, south-east, south-west, north-west), according to our experimental questions. For each category, separately for each domain, we excluded distances that were below or above two standard deviations from the average distance (across subjects and pairs of stimuli).

#### Experimental questions and statistical comparisons

**Fig. 2:**
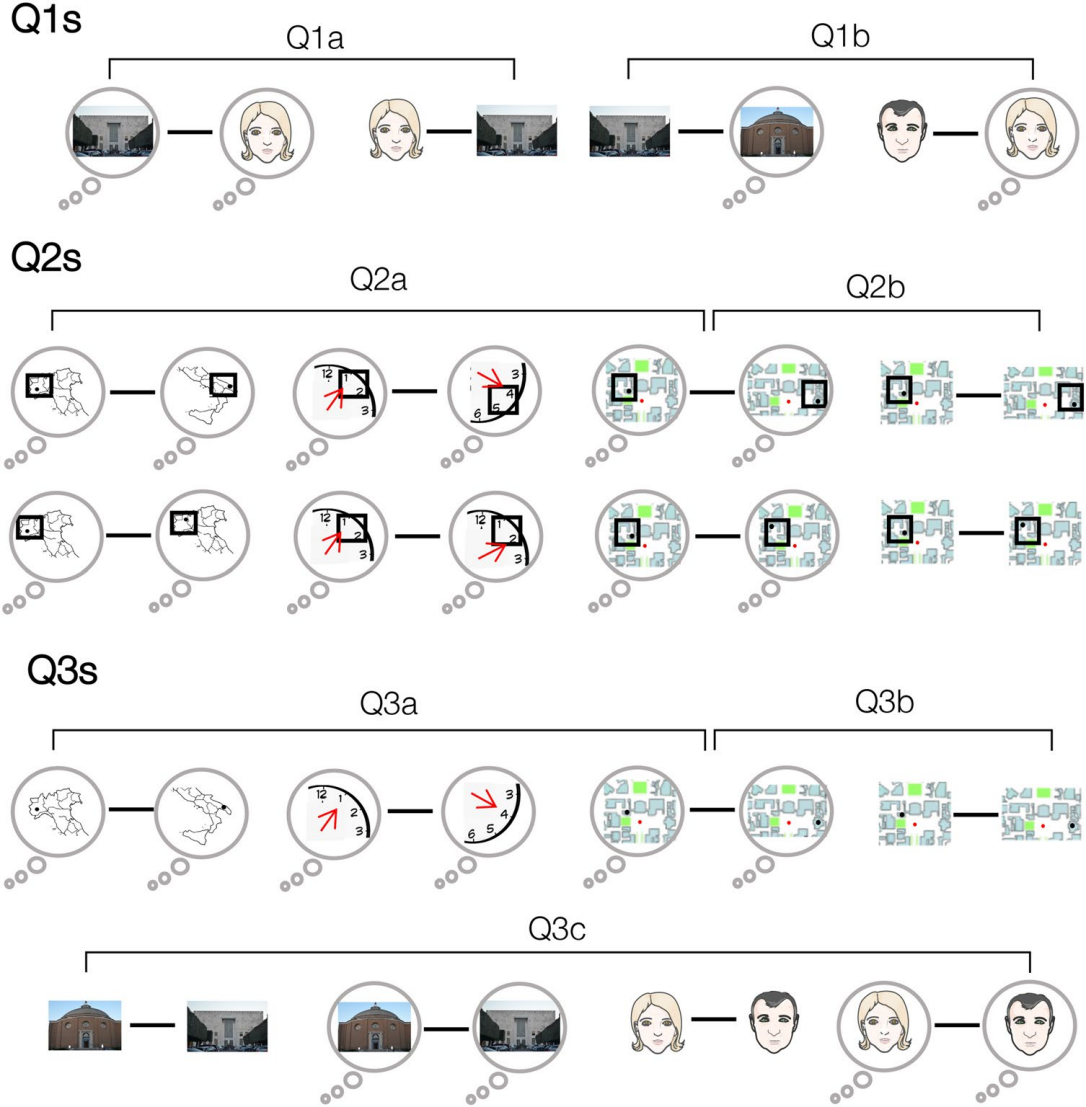
Schematic illustration of the three experimental questions (Q1s, Q2s and Q3s), along with their specific subquestions. The figure shows a graphical representation of each pair of stimuli averaged according to each experimental question. Clouds around stimuli distinguish the visual mental images from the perceived stimuli. At difference with Experiments 1 and 2, in Experiment 3, subjects did not recall the spatial location of the stimuli; accordingly, buildings are displayed as pictures in Q3c and Q1s, as points on a map in Q2s. The first row of the figure represents the preliminary analyses (Q1s) performed by averaging pairs of faces and buildings, separately for each domain (Q1a), and averaging pairs of perceived and imagined stimuli, separately for each category (Q1b). The third and the fourth rows graphically display the Q2s questions, relative to the dissimilarity between pairs of stimuli belonging to different quadrants (the third row), or to the same quadrant (the fourth row), separately for each category (i.e., city, hour and building, Q2a) and domain (imagery or perception, Q2b). Stimuli are represented as black dots located in the map of Italy, on a clock, or in the map of the campus; the black squares around the dots provide the spatial information (i.e., the belonging quadrant) of each exemplar. Differently, since the Q3s questions were independent from the spatial location of the stimuli, no black square around the exemplars is shown in Q3a, relative to imagined cities, hours and buildings, and in Q3b, relative to imagined and perceived buildings. The last row shows a graphical representation of the Q3c question, analyzed by averaging pairs of buildings and of faces, separately for each domain

Subsequent comparisons were aimed at addressing three sets of experimental questions, resulting in seven specific subquestions that are listed below and whose aims are detailed in the *Results* section. For a graphical representation of each question and subquestion, see Fig. 2.

- Q1a: Is the discriminability between faces and buildings higher during perception than imagery?
- Q1b: Is the discriminability between imagined and perceived stimuli different between categories, reflecting the regional preference in the HVC?
- Q2a: Is spatial information of topological and non-topological mental images coded within the HVC and the HC?
- Q2b: Are spatial positions of buildings encoded better during perception than during imagery?
- Q3a: Are topological mental images coded better than non-topological ones within the HVC?
- Q3b: Are buildings encoded better during perception than during imagery?
- Q3c: Is the amount of similarity between exemplars higher during perception than during imagery, and different across buildings and faces?

The first set of questions (Q1s) were conceived as a collection of preliminary analyses aimed at verifying the method we implemented here. To this aim, we tested inter-category (i.e., faces and landmarks) and inter-domain (i.e., imagery and perception) dissimilarities, by averaging pairs of exemplars belonging to different categories, separately for each domain (Q1a), or to different domains, separately for each category (Q1b). For Q1 questions, only data from Experiment 3 were tested, since Experiments 1 and 2 did not include both face and landmark categories.

The second set of questions (Q2s) were focused on the encoding of the spatial information of topological and non-topological mental images (Q2a), and of topological stimuli in both domains (i.e., imagery and perception; Q2b). To address these questions, only data from Experiments 1 and 2 were tested, since we did not ask participants to imagine the spatial position of buildings during the Experiment 3; furthermore, we collapsed data from Experiments 1 and 2 for the building category, whereas we used only data from Experiment 1 for the other categories (i.e., cities and hours). On a side note, since 2 individuals participated in both experiments 1 and 2, their data were averaged across experiments for imagined buildings.

The third set of questions (Q3s) aimed at analyzing the more subtle intra-category (i.e., between different exemplars of stimuli belonging to the same category) and intra-domain (i.e., between different exemplars of stimuli belonging to the same domain) discrimination. In Q3a and Q3b, we used only data from Experiments 1 and 2, similarly as in Q2; instead, for Q3c question, we tested only data from Experiment 3, in both scene- and face-selective regions, to test for possible distributed encoding in the HVC. Furthermore, even if both Q3b and Q3c analyzed the difference among perceived and imagined landmarks, we addressed these questions separately to avoid the spurious effect of the different tasks used in the Experiments 2 and 3.

For each question, we first performed one-sample *t*-tests to check whether average distances were higher than zero in the areas of HVC and in the HC, i.e., to test whether these regions were able to discriminate pairs of exemplars selected according to each experimental subquestion. When an area significantly encoded the dissimilarity in more than one category or domain, as a second step, we checked whether in that area there was a better discrimination in one category or domain over the others, by performing paired or two-sample *t*-tests (i.e., two categories) or ANOVA (more than two categories), according to our experimental hypotheses (for similar procedures, see Beukema et al. 2019; Yokoi et al. 2018).

For each analysis, significance level was set at *p* < 0.05, adjusted using Bonferroni’s correction for multiple comparisons at the seed level (i.e., the number of seed regions included in each analysis). Thus, for one-sample *t*-tests, significance level was set at *p* < 0.00625 in the analyses on data from Experiments 1 and 2 (i.e., Q2 questions, Q3a question and Q3b question) since these analyses were performed only on the scene-selective regions of the HVC and on the HC, whereas it was set at *p* < 0.00416 in the analyses on data from Experiment 3 (i.e., Q1 questions and Q3c question) that were performed also on the face-selective areas in the HVC. Similarly, significance level of the second level analyses (i.e., ANOVAs, paired or two-sample *t*-tests) was set using Bonferroni’s correction for multiple comparisons, depending on the number of regions included in each analysis (more details are provided in the result section).

### Data visualization

**Fig. 3:**
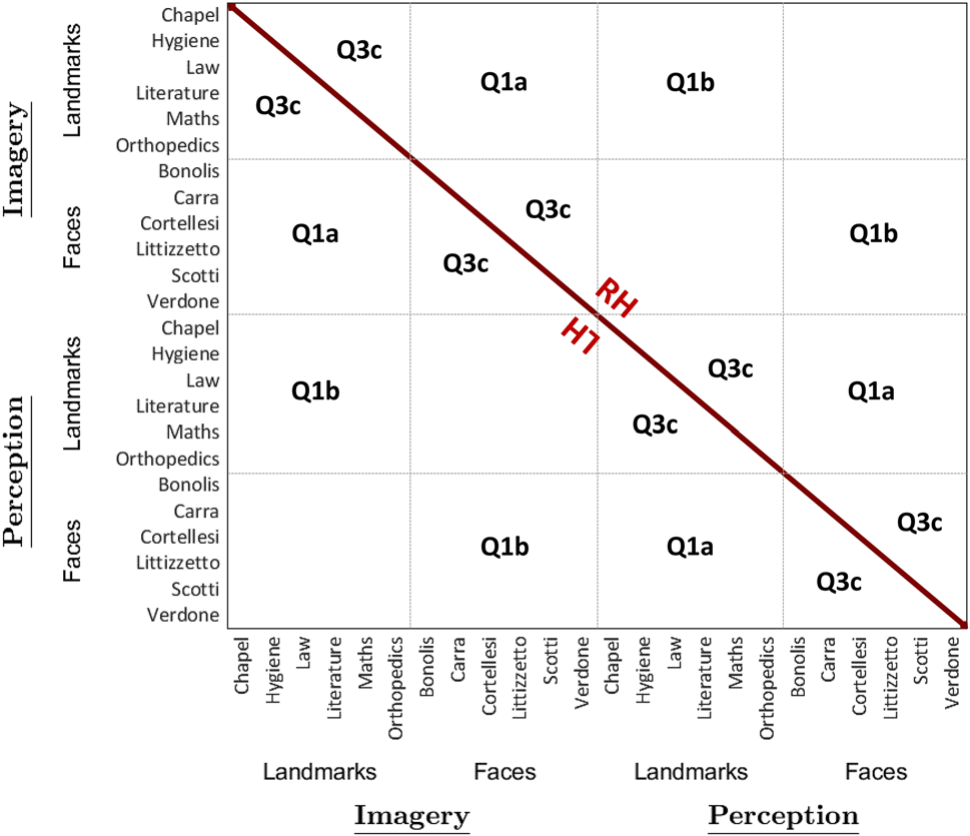
Schematic representation of the representational dissimilarity matrices of Q1s and Q3c questions. To increase readability of the representational dissimilarity matrices displayed in Fig. 4, we provided a schematic matrix summarizing experimental questions (Q1a, Q1b and Q3c)

**Fig. 4:**
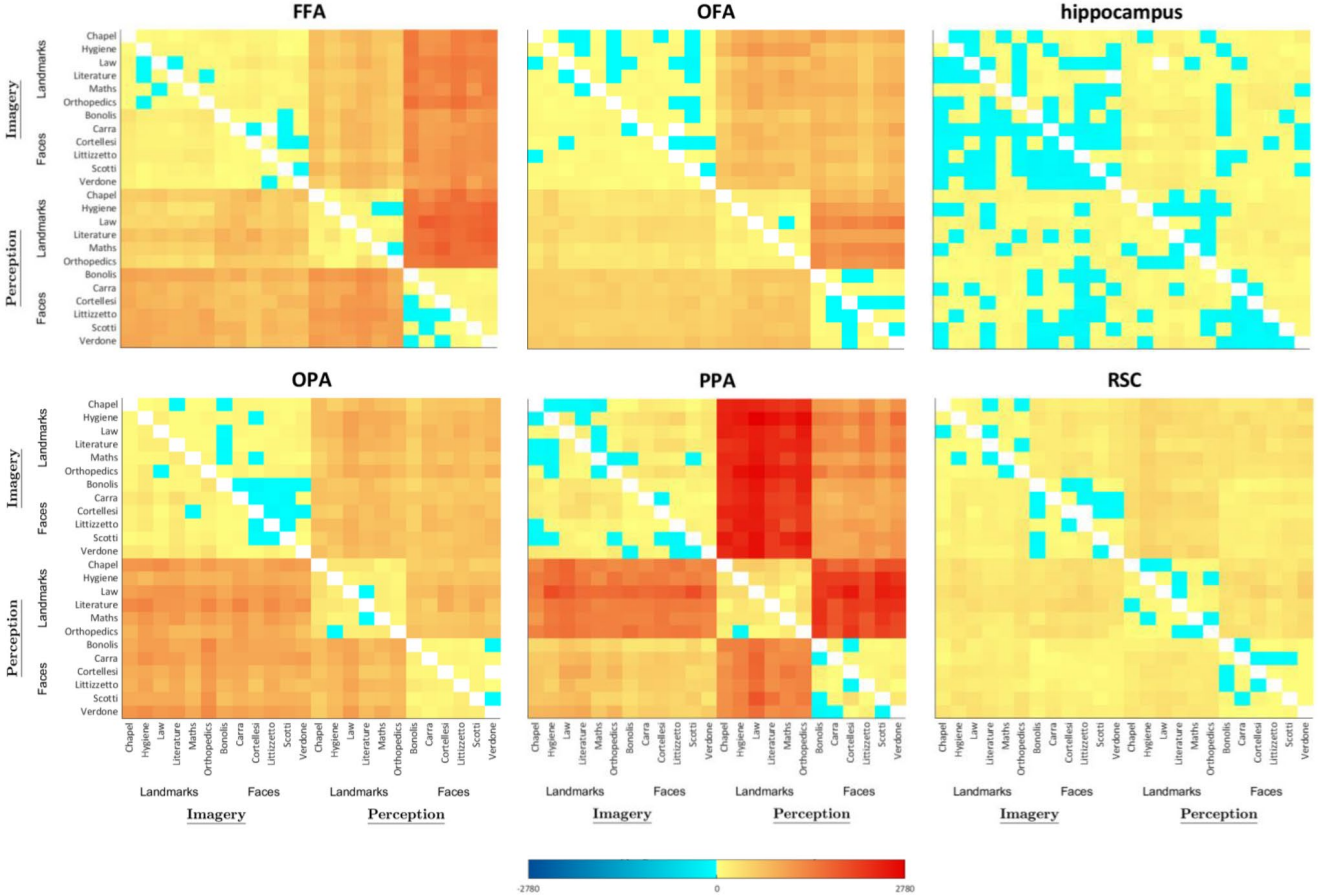
Representational Dissimilarity Matrices of Q1 questions and Q3c question- Imagined and perceived faces and buildings. Mean crossnobis distances between each pair of exemplars are plotted in turquoise to dark blue, if negative (range: from − 1 to − 2780), and in pale yellow to dark red if positive (range: from 1 to 2780); distances equal to zero (i.e. in the leading diagonal) are plotted in white. Matrix elements below the main diagonal represent the left hemisphere results, whereas those above the main diagonal represent the right hemisphere results

**Fig. 5:**
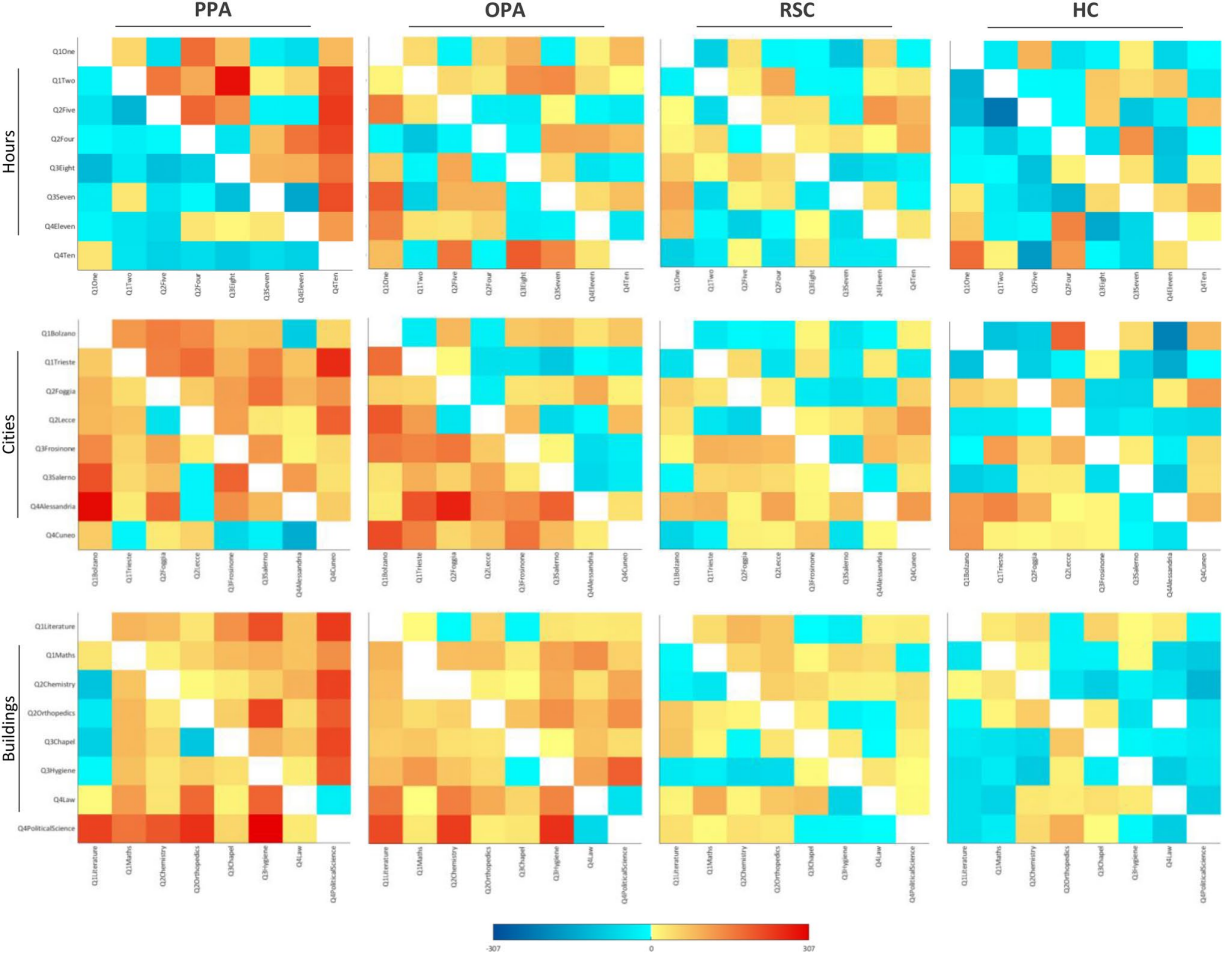
Representational Dissimilarity Matrices of Q2a and Q3a questions- Imagined hours, cities and buildings, and the spatial location of each stimulus. For each exemplar, the name and the spatial location are provided, by indicating the belonging quadrant (Q1, Q2, Q3 or Q4). Mean crossnobis distances between each pair of exemplars are plotted in turquoise to dark blue, if negative (range: from − 1 to − 307), and in pale yellow to dark red if positive (range: from 1 to 307); distances equal to zero (i.e., in the leading diagonal) are plotted in white. Matrix elements below the main diagonal represent the left hemisphere results, whereas those above the main diagonal represent the right hemisphere results. The diagonal below the leading diagonal represents mean distances of pairs of exemplars belonging to the same quadrant in the left hemisphere; the diagonal upper the leading diagonal represents mean distances of pairs of exemplars belonging to the same quadrant in the right hemisphere

**Fig. 6:**
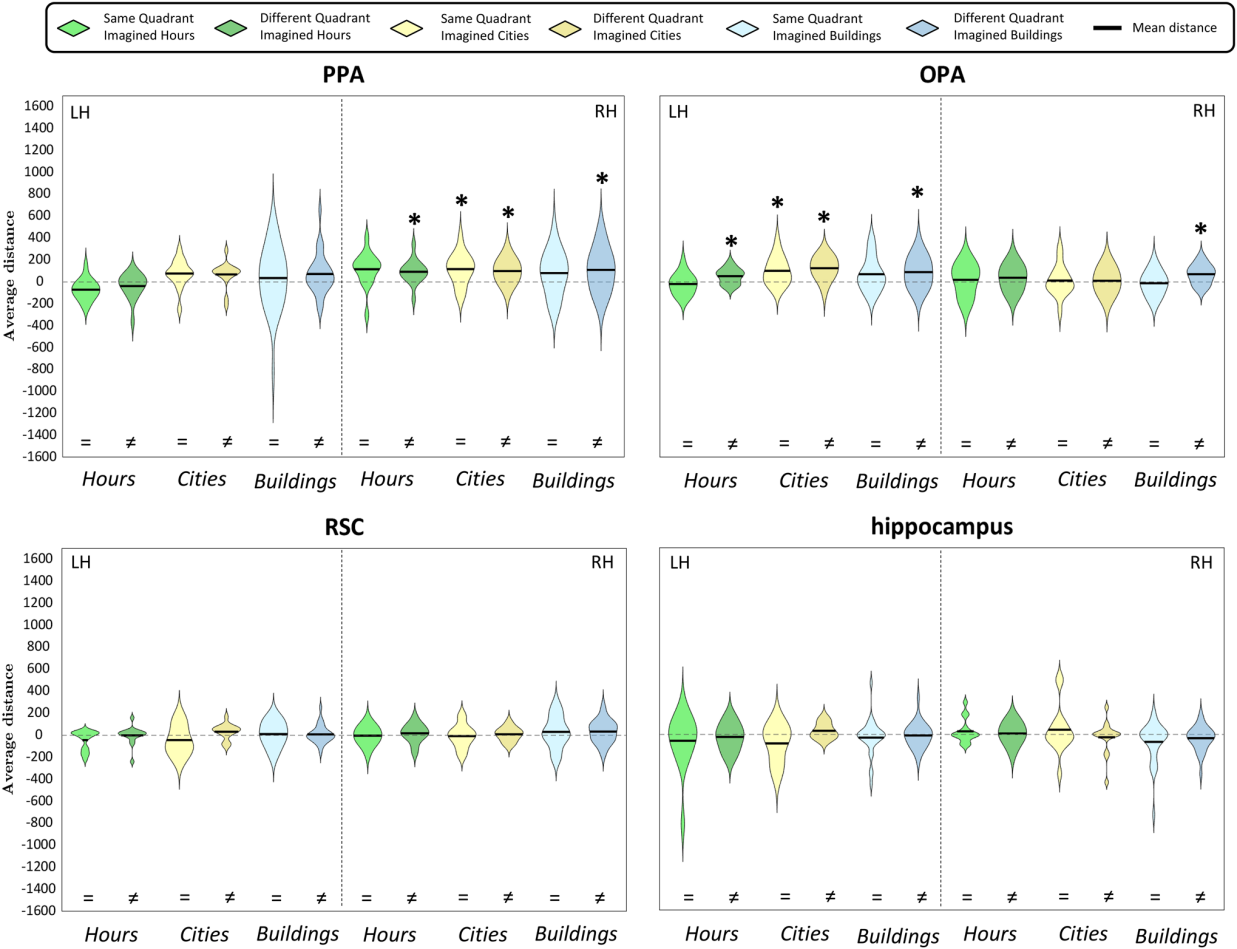
Violin plots of Q2a question- Distribution across subjects of the mean distances between pairs of imagined hours, cities and buildings, separately for spatial location (same or different quadrants). For each region, we plotted mean distances separately for each category and spatial location; the black dashed vertical line splits the violin plots of the left (LH) and the right (RH) hemispheres. Results are plotted in a range from − 1600 to 1700. Black asterisks above the violins show significance of the one-sample *t*-tests (*p* < 0.05, corrected for multiple comparisons)

**Fig. 7:**
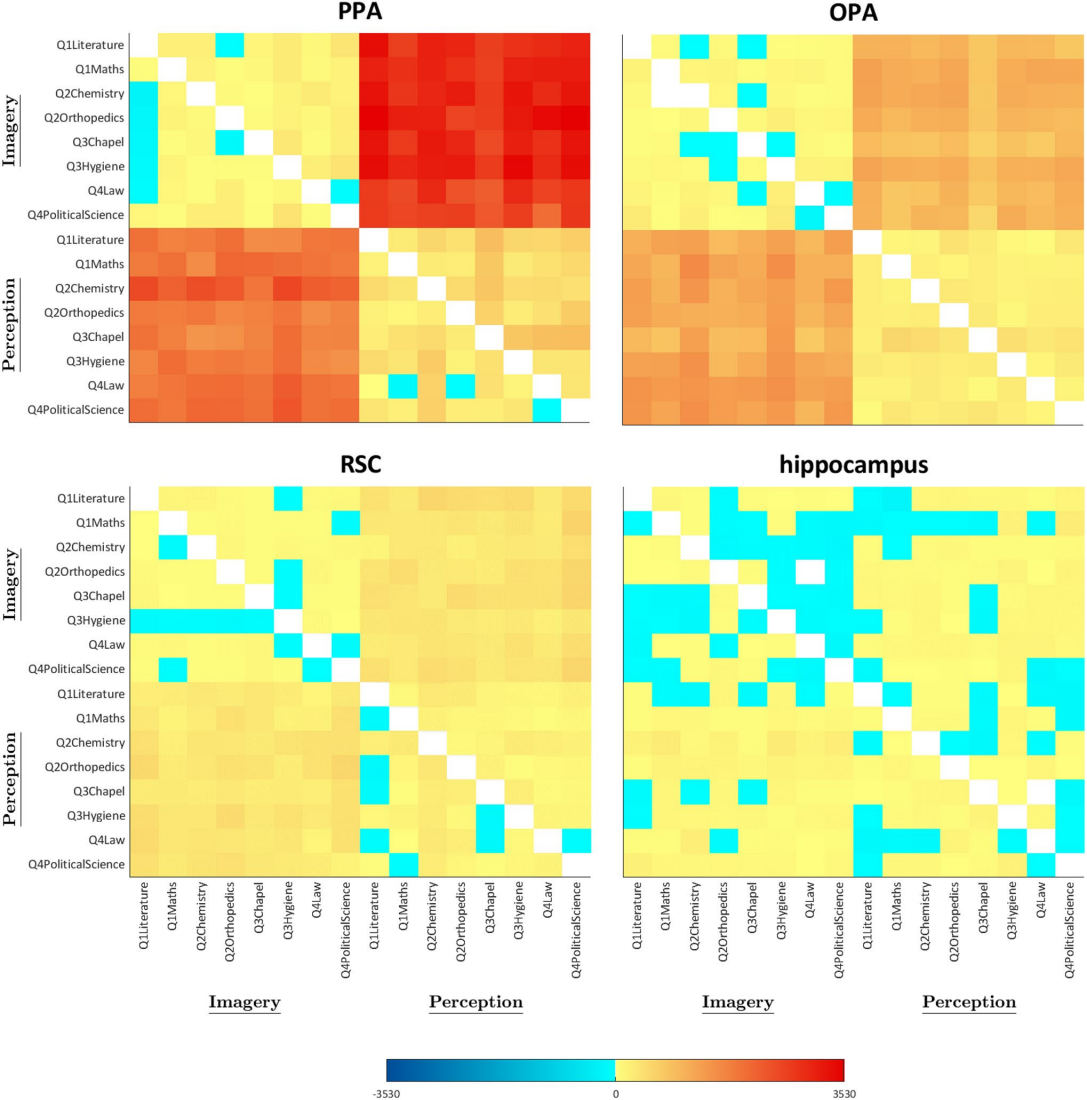
Representational Dissimilarity Matrices of Q2b and Q3b questions- Imagined and perceived buildings, and the spatial location of each stimulus. For each exemplar, the name and the spatial location are provided, by indicating the belonging quadrant (Q1, Q2, Q3 or Q4). Mean crossnobis distances between each pair of exemplars are plotted in turquoise to dark blue, if negative (range: from −1 to − 3530), and in pale yellow to dark red if positive (range: from 1 to 3530); distances equal to zero (i.e. in the leading diagonal) are plotted in white. Matrix elements below the main diagonal represent the left hemisphere results, whereas those above the main diagonal represent the right hemisphere results. The diagonal below the leading diagonal represents mean distances of pairs of exemplars belonging to the same quadrant in the left hemisphere; the diagonal upper the leading diagonal represents mean distances of pairs of exemplars belonging to the same quadrant in the right hemisphere. Distances between pairs of imagined buildings are the same provided in the Fig. 5, but the range is different to compare them to the distances between pairs of perceived buildings

**Fig. 8:**
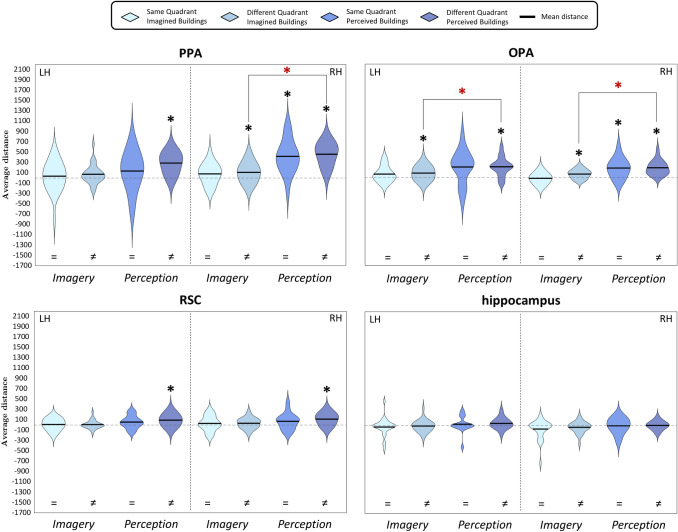
Violin plots of Q2b question- Distribution across subjects of the mean distances between pairs of imagined and perceived buildings, separately for spatial location (same or different quadrants). For each region, we plotted mean distances separately for each domain and spatial location; the black dashed vertical line splits the violin plots of the left (LH) and the right (RH) hemispheres. Results are plotted in a range from − 1700 to 2200; results of the imagery domain are the same as shown in Fig. 6, but here are shown in a different range to compare them to the perception domain. Black asterisks above the violins show significance of the one-sample *t*-tests (*p* < 0.05, corrected for multiple comparisons). Black lines above the violins also show the comparisons performed by mean of paired or two-sample *t*-tests (for more details, see Methods); dark red asterisks above those lines indicate significant differences between domains and/or spatial locations, as resulting from the paired or two-sample *t*-tests (*p* < 0.05, corrected for multiple comparisons)

**Fig. 9:**
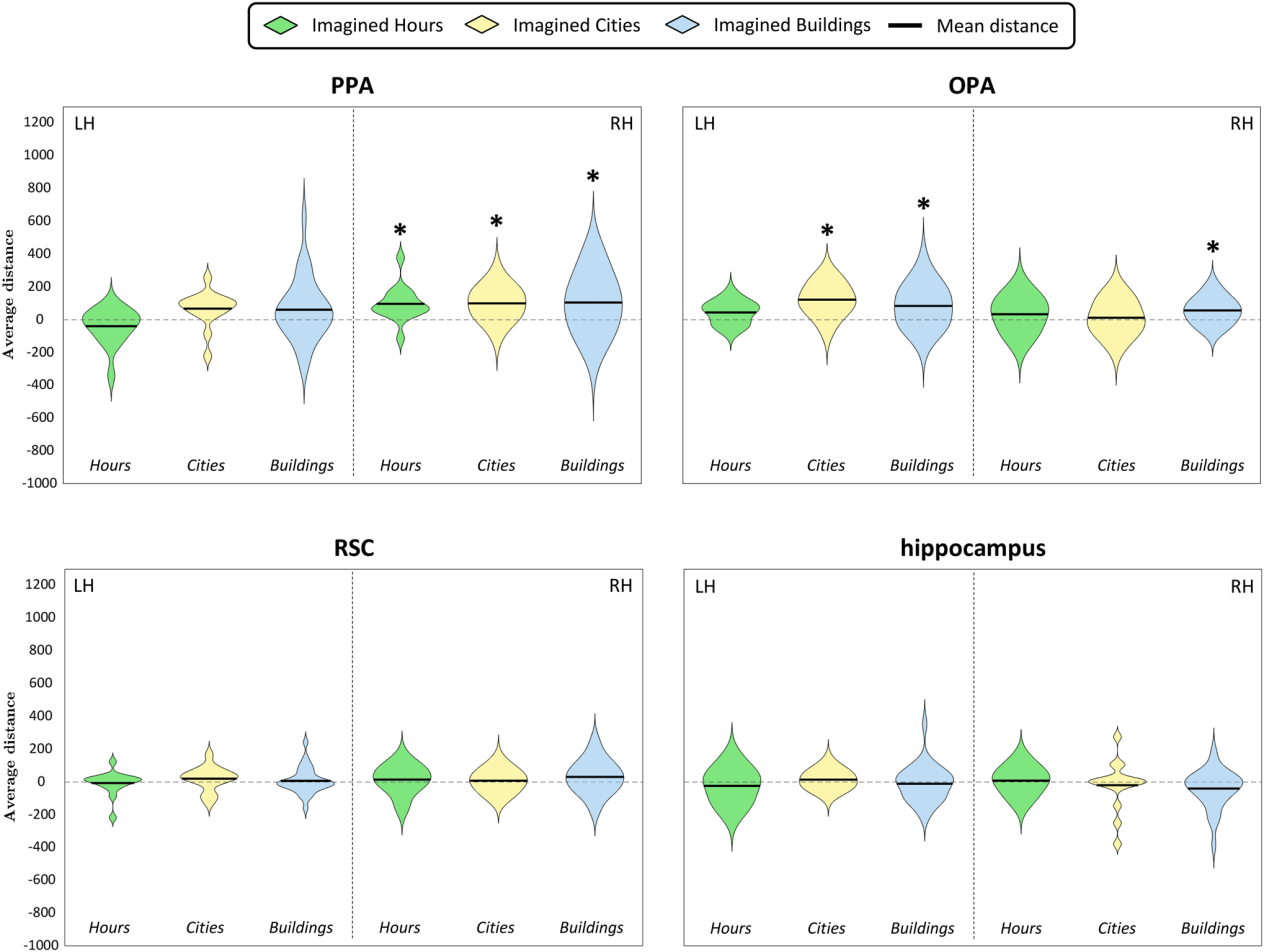
Violin plots of Q3a question- Distribution across subjects of the mean distances between pairs of imagined hours, cities and buildings. For each region, we plotted mean distances of all categories; the black dashed vertical line splits the violin plots of the left (LH) and the right (RH) hemispheres. Results are plotted in a range from − 1000 to 1300. Black asterisks above the violins show significance of the one-sample *t*-tests (*p* < 0.05, corrected for multiple comparisons)

**Fig. 10:**
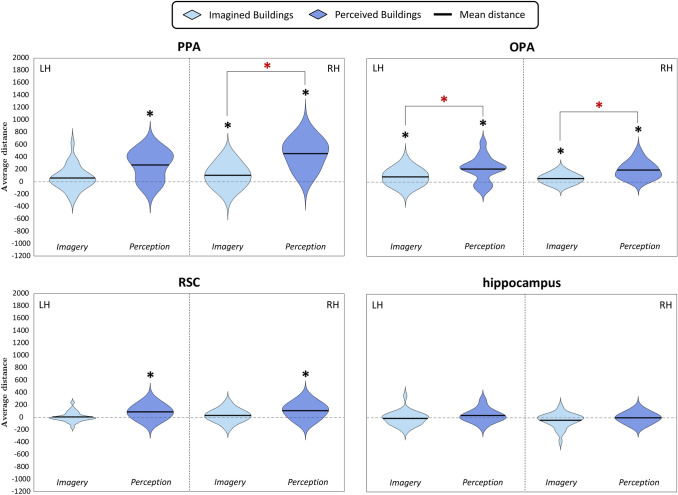
Violin plots of Q3b question- Distribution across subjects of the mean distances between pairs of imagined and perceived buildings. For each region, we plotted mean distances of both domains; the black dashed vertical line splits the violin plots of the left (LH) and the right (RH) hemispheres. Results are plotted in a range from − 1200 to 2000; results of the imagery domain are the same as shown in Fig. 7, but here are shown in a different range to compare them to the perception domain. Black asterisks above the violins show significance of the one-sample *t*-tests (*p* < 0.05, corrected for multiple comparisons). Black lines above the violins also show the comparisons performed by mean of paired or two-sample *t*-tests (for more details, see Methods); dark red asterisks above those lines indicate significant differences between domains, as resulting from the paired or two-sample *t*-tests (*p* < 0.05, corrected for multiple comparisons)

**Fig. 11:**
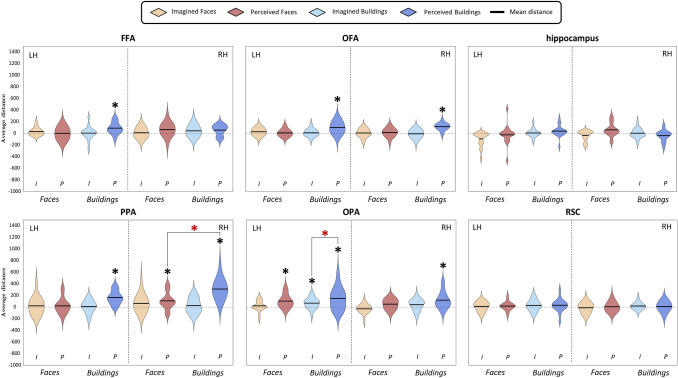
Violin plots of Q3c question- Distribution across subjects of the mean distances between pairs of imagined and perceived buildings, and imagined and perceived faces. For each region, we plotted mean distances of both domains and categories; the black dashed vertical line splits the violin plots of the left (LH) and the right (RH) hemispheres. Results are plotted in a range from − 1000 to 1500; Black asterisks above the violins show significance of the one-sample *t*-tests (*p* < 0.05, corrected for multiple comparisons). Black lines above the violins also show the comparisons performed by mean of paired or two-sample *t*-tests (for more details, see Methods); dark red asterisks above those lines indicate significant differences between conditions or domains, as resulting from the paired or two-sample *t*-tests (*p* < 0.05, corrected for multiple comparisons). *P* perception, *I* imagery

To display the results of our analyses, we plotted RDMs to visualize distances between pairs of exemplars, and we used violin plots (Hoffmann 2015) to visualize the distribution across subjects of average distances, separately for each category and domain, and according to each experimental question. First, we include a schematic representation (Fig. 3) of the RDMs representing the Q1a, Q1b and Q3c questions to increase the readability of the corresponding RDMs, depicted in Fig. 4. Following figures (from Figs. 5, 6, 7, 8, 9, 10 and 11) show the RDMs and the violin plots, separately for each question and subquestion.

## Results

### Preliminary analyses (Q1s)

Since this is the very first study using the crossnobis estimator to perform a RSA on imagined stimuli, we aimed at verifying its reliability in replicating previous findings using different estimators (Boccia et al. 2017). Thus, we conceived the first set of questions (Q1a and Q1b) as a collection of preliminary analyses aimed at testing inter-category (i.e., faces and landmarks) and inter-domain (i.e., imagery and perception) dissimilarities. Consistent with the previous studies (Cichy et al. 2012; Haxby et al. 2001; Ishai et al. 1999; Lee et al. 2012; O’Toole et al. 2005; Reddy et al. 2010), we hypothesized that all the areas in the HVC and the HC were able to discriminate faces from buildings (Q1a) and perceived stimuli from imagined ones (Q1b).

#### Q1a: Is the discriminability between faces and buildings higher during perception than imagery?

As a first step, we tested the discriminability between faces and buildings separately for the perception and the imagery domain, by performing one-sample *t*-tests on average neural dissimilarities between pairs of exemplars (Fig. 4) belonging to different categories (i.e., faces and buildings), resulting in two analyses for each region. Results of the one-sample *t*-tests showed that all the areas in the HVC, plus the bilateral HC, discriminated perceived faces and perceived buildings (FFA LH: *t*_18_ = 6.98, *p* = 8.11 × 10^–7^; FFA RH: *t*_18_ = 9.01, *p* = 2.17 × 10^–8^; OFA LH: *t*_18_ = 7.31, *p* = 4.37 × 10^–7^; OFA RH: *t*_18_ = 7.63, *p* = 2.38 × 10^–7^; OPA LH: *t*_18_ = 7.17, *p* = 5.59 × 10^–7^; OPA RH: *t*_18_ = 8.32, *p* = 7.01 × 10^–8^; PPA LH: *t*_18_ = 11.81, *p* = 3.24 × 10^–8^; PPA RH: *t*_18_ = 10.59, *p* = 1.83 × 10^–8^; RSC LH: *t*_18_ = 6.24, *p* = 3.74 × 10^–6^; RSC RH: *t*_18_ = 7.65, *p* = 2.31 × 10^–7^; HC LH: *t*_18_ = 3.46, *p* = 1.38 × 10^–3^; HC RH: *t*_18_ = 3.79, *p* = 6.69 × 10^–4^). Instead, imagined faces and imagined buildings are discriminated by all the areas in the HVC except for the right OPA (FFA LH: *t*_18_ = 4.75, *p* = 8.04 × 10^–5^; FFA RH: *t*_18_ = 5.47, *p* = 1.71 × 10^–5^; OFA LH: *t*_18_ = 3.14, *p* = 2.82 × 10^–3^; OFA RH: *t*_18_ = 3.13, *p* = 2.93 × 10^–3^; PPA LH: *t*_18_ = 5.83, *p* = 8.08 × 10^–6^; PPA RH: *t*_18_ = 3.94, *p* = 4.75 × 10^–4^; RSC LH: *t*_18_ = 4.02, *p* = 3.99 × 10^–4^; RSC RH: *t*_18_ = 6.11, *p* = 4.56 × 10^–6^; OPA LH: *t*_18_ = 5.74, *p* = 9.58 × 10^–6^).

We then performed one-tailed paired *t*-tests on the areas that were able to detect dissimilarity between faces and buildings in both domains (i.e., the bilateral FFA, OFA, PPA, the left OPA and the right RSC), hypothesizing that distances in the perception domain would be higher than distances in the imagery domain. Results of the one-tailed paired *t*-tests (perception > imagery, *p* < 0.0056) showed that the bilateral FFA (LH: *t*_18_ = 6.28, *p* = 3.21 × 10^–6^; RH: *t*_18_ = 8.91, *p* = 2.61 × 10^–8^), the bilateral OFA (LH: *t*_18_ = 7.19, *p* = 5.42 × 10^–7^; RH: *t*_18_ = 7.55, *p* = 2.75 × 10^–7^), the bilateral PPA (LH: *t*_18_ = 10.67, *p* = 1.64 × 10^–9^; RH: *t*_18_ = 9.52, *p* = 9.44 × 10^–9^), the left OPA (*t*_18_ = 5.74, *p* = 9.69 × 10^–6^) and the right RSC (*t*_18_ = 4.12, *p* = 3.21 × 10^–4^) discriminated better perceived than imagined pairs of exemplars belonging to different categories. Only a trend towards significance was detected in the left RSC (*t*_18_ = 2.81, *p* = 5.73 × 10^–3^).

In sum, all the regions of the HVC and the HC discriminated between faces and buildings during perception. However, during imagery, exemplars belonging to different categories were discriminated by later visual areas (FFA, PPA, RSC) and early visual areas with the exception of right OPA (i.e., bilateral OFA and left OPA). Bilateral FFA, OFA and PPA, as well as left OPA and right RSC, discriminated exemplars of different categories better during perception than imagery.

#### Q1b: Is the discriminability between imagined and perceived stimuli different between categories, reflecting the regional preference in the HVC?

We first tested the discriminability between imagined and perceived stimuli separately for each category (i.e., faces and buildings), by performing one-sample *t*-tests on average neural dissimilarities (Fig. 4) between pairs of exemplars belonging to different domains (i.e., perception and imagery), resulting in two analyses for each region. We found that all the areas in the HVC (FFA LH: *t*_18_ = 5.71, *p* = 1.03 × 10^–5^; FFA RH: *t*_18_ = 7.56, *p* = 2.71 × 10^–7^; OFA LH: *t*_18_ = 6.21, *p* = 3.67 × 10^–6^; OFA RH: *t*_18_ = 9.36, *p* = 1.23 × 10^–8^; OPA LH: *t*_18_ = 5.37, *p* = 2.11 × 10^–5^; OPA RH: *t*_18_ = 7.61, *p* = 2.47 X 10^–7^; PPA LH: *t*_18_ = 10.67, *p* = 1.63 × 10^–9^; PPA RH: *t*_18_ = 12.42, *p* = 1.46 × 10^–10^; RSC LH: *t*_18_ = 7.89, *p* = 1.51 × 10^–7^; RSC RH: *t*_18_ = 7.97, *p* = 1.28 × 10^–7^), and the bilateral HC (LH: *t*_18_ = 3.47, *p* = 1.36 × 10^–3^; RH: *t*_18_ = 3.07, *p* = 3.33 × 10^–3^), discriminated between imagined and perceived buildings, whereas all areas in the HVC (FFA LH: *t*_18_ = 7.12, *p* = 6.14 × 10^–7^; FFA RH: *t*_18_ = 7.26, *p* = 4.77 × 10^–7^; OFA LH: *t*_18_ = 7.79, *p* = 1.81 × 10^–7^; OFA RH: *t*_18_ = 7.97, *p* = 1.93 × 10^–7^; OPA LH: *t*_18_ = 5.27, *p* = 2.59 × 10^–5^; OPA RH: *t*_18_ = 7.86, *p* = 1.56 × 10^–7^; PPA LH: *t*_18_ = 8.69, *p* = 3.66 × 10^–8^; PPA RH: *t*_18_ = 10.84, *p* = 1.28 × 10^–9^; RSC LH: *t*_18_ = 5.08, *p* = 3.88 × 10^–5^; RSC RH: *t*_18_ = 6.53, *p* = 1.93 × 10^–6^), but not the bilateral HC, discriminated between imagined and perceived faces.

Our hypothesis was that dissimilarities between imagined and perceived stimuli would be higher in the face category than in the building category in face-selective areas, whereas dissimilarities between imagined and perceived stimuli would be higher in the building category than in the face category in scene-selective areas. This finding was predicted based on the regional category-specific preference in the HVC, and on the stronger discrimination of individual objects during perception than imagery found by Lee et al. (2012). Thus, we performed one-tailed paired *t*-tests separately for face-selective areas (i.e., OFA and FFA) and scene-selective areas (i.e., OPA, PPA and RSC). Results of the one-tailed paired *t*-tests in the face-selective areas (faces > buildings, *p* < 0.0125) showed significant differences in the left OFA (*t*_18_ = 2.49, *p* = 1.12 × 10^–2^) and the right FFA (*t*_18_ = 4.35, *p* = 1.91 × 10^–4^); results of the one-tailed paired *t*-tests in scene-selective areas (buildings > faces, *p* < 0.0083) showed significant differences in the bilateral PPA (LH: *t*_18_ = 9.16, *p* = 1.68 × 10^–8^; RH: *t*_18_ = 10.27, *p* = 2.94 × 10^–9^) and RSC (LH: *t*_18_ = 4.95, *p* = 5.12 × 10^–5^; RH: *t*_18_ = 5.04, *p* = 4.24 × 10^–5^), but not in the bilateral OPA.

In sum, exemplars belonging to different domains were widely discriminated across the areas in the HVC in both categories, whereas the bilateral HC only discriminated between imagined and perceived buildings. Among face-selective regions, only the left OFA and the right FFA discriminated stimuli belonging to different domains better in the face category than in the building category. Instead, buildings of different domains were discriminated better than faces of different domains in the later visual areas (i.e., the bilateral PPA and RSC), but not in the bilateral OPA. The RDM corresponding to the Q1s is reported in Fig. 4.

Overall Q1s, as a set of preliminary analyses, supports previous results and hypotheses (O’Toole et al. 2005; Haxby et al. 2001) and, thus, provides support for the method we implemented here.

### Spatial coding of topological and non-topological stimuli in the HVC and the HC (Q2s)

Q2s questions aimed at unveiling whether the HVC and the HC encode the spatial information, operationalized in terms of quadrants, of topological and non-topological mental images (Q2a), and of topological stimuli in both domains (i.e., imagery and perception; Q2b). Indeed, exemplars belonging to the same quadrant were supposed to share the same spatial information, contrary to the exemplars belonging to different quadrants; thus, we averaged distances of different exemplars, depending on the quadrant (same or different). Based on previous literature (Boccia et al. 2015, 2017; Sulpizio et al. 2014) and due to our focus on scene-selective regions in the HVC, we predicted that topological mental images were better discriminated than non-topological mental images (Q2a). Based on a previous study, finding that the pattern of dissimilarities between individual objects was higher during perception than imagery in all the regions of the ventral visual stream (Lee et al. 2012), we further hypothesized that the discrimination was better when the spatial information was different, and that the specificity of neural representation would be emphasized during perception (Q2b).

#### Q2a: Is spatial information of topological and non-topological mental images coded within the HVC and the HC?

As a first step, we checked whether areas within the HVC and HC encode the neural dissimilarities (Fig. 5) between pairs of imagined exemplars belonging to the same or to different quadrants, separately for each category. To this aim, we performed separate one-sample *t*-tests for each category (i.e., buildings, cities on the map of Italy and hours on the clock) and spatial location (same quadrant or different quadrants), resulting in six one-sample *t*-tests for each region (Fig. 6). We found that exemplars belonging to the same quadrant were discriminated only in the city category by the left OPA (*t*_14_ = 3.12, *p* = 3.74 × 10^–3^) and the right PPA (*t*_14_ = 3.13, *p* = 3.66 × 10^–3^). Instead, the left OPA and the right PPA discriminated pairs of exemplars belonging to different quadrants both in the city category (OPA LH: *t*_14_ = 4.68, *p* = 1.76 × 10^–4^; PPA RH: *t*_14_ = 3.46, *p* = 1.91 × 10^–3^) and the hour category (OPA LH: *t*_14_ = 2.96, *p* = 5.21 × 10^–3^; PPA RH: *t*_14_ = 3.29, *p* = 2.69 × 10^–3^). Dissimilarities between pairs of buildings belonging to different quadrants were significantly higher than zero in the bilateral OPA (LH: *t*_28_ = 3.37, *p* = 1.11 × 10^–3^; RH: *t*_28_ = 4.32, *p* = 8.88 × 10^–5^) and the right PPA (*t*_28_ = 3.02, *p* = 2.64 × 10^–3^).

We then performed paired *t*-tests on the left OPA and the right PPA (significance level: *p* < 0.025) to test whether distances between cities belonging to different quadrants were higher than distances between cities belonging to the same quadrant. Results did not show significant differences, neither in left OPA nor in right PPA (*p*_s_ > 0.228).

Also, since the left OPA and the right PPA discriminated pairs of exemplars belonging to different quadrants in all categories, we performed one-way ANOVAs (significance level: *p* < 0.025) to test possible differences between categories; no significant difference was detected (*p*_s_ > 0.140).

In sum, exemplars belonging to the same quadrant were discriminated only in the city category by the left OPA and the right PPA, to the same extent of cities belonging to different quadrants. The left OPA and the right PPA also discriminated between exemplars belonging to different quadrants in all categories to the same extent; instead, the right OPA discriminated only between pairs of buildings belonging to different quadrants.

#### Q2b: Are spatial positions of buildings encoded better during perception than during imagery?

We addressed this question by checking whether neural representations (Fig. 7) associated with buildings located in the same quadrant were the same or different to those associated with buildings located in different quadrants, separately for each domain.

One-sample *t*-tests were performed on the average neural dissimilarities of exemplars belonging to the same quadrant or to different quadrants in the perception domain, resulting in two separate analyses for each region (Fig. 8). Results of one-sample *t*-tests showed that exemplars belonging to the same quadrant were encoded in the right OPA (*t*_15_ = 3.82, *p* = 8.39 × 10^–4^) and the right PPA (*t*_15_ = 5.13, *p* = 6.16 × 10^–5^), whereas exemplars belonging to different quadrants were encoded in the bilateral OPA (LH: *t*_15_ = 4.57, *p* = 1.82 × 10^–4^; RH: *t*_15_ = 5.67, *p* = 2.23 × 10^–5^), the bilateral PPA (LH: *t*_15_ = 5.79, *p* = 1.76 × 10^–5^; RH: *t*_15_ = 8.12, *p* = 3.61 × 10^–7^), and the bilateral RSC (LH: *t*_15_ = 3.34, *p* = 2.25 × 10^–3^; RH: *t*_15_ = 3.77, *p* = 9.33 × 10^–4^).

We further predicted that the dissimilarities between exemplars belonging to different quadrants were higher than between exemplars belonging to the same quadrant in the right OPA and the right PPA. We disentangled that by performing a one-tailed paired *t*-test (significance level: *p* < 0.025), which did not show significant differences (*p*_s_ > 0.238).

As a second step, we compared these data to the data resulting from the Q2a question relative to the building category in the imagery domain (i.e., the dissimilarity between imagined buildings belonging to the same quadrant or to different quadrants). Since no region encoded the dissimilarity among exemplars of topological mental images belonging to the same quadrant (Q2a), we performed further domain-related analyses only on pairs of buildings belonging to different quadrants, hypothesizing that the dissimilarity would be higher during perception than during imagery. A direct comparison between the perception and the imagery domain in the bilateral OPA and the right PPA (significance level: *p* < 0.016) by means of two-sample *t*-tests showed that neural dissimilarities were higher between perceived buildings belonging to different quadrants than between imagined buildings belonging to the different quadrants in all the areas (OPA LH: *t*_27_ = 2.46, *p* = 8.89 × 10^–3^; OPA RH: *t*_27_ = 3.65, *p* = 3.54 × 10^–4^; PPA RH: *t*_27_ = 5.44, *p* = 1.17 × 10^–6^).

In brief, we found that during perception, only the right OPA and the right PPA discriminated between exemplars belonging to the same quadrant; instead, all the scene-selective regions of the HVC, but not the bilateral HC, discriminated exemplars from different quadrants. Furthermore, the right OPA and the right PPA discriminated between pairs of perceived buildings belonging to the same quadrant and to different quadrants to the same extent. The bilateral OPA and in the right PPA discriminated perceived buildings belonging to different quadrants better than imagined buildings belonging to the different quadrants.

### Coding of topological and non-topological exemplars within the HVC and the HC (Q3s)

At difference with Q2s, here we focused on neural dissimilarities between topological and non-topological exemplars, independent of their spatial information. We hypothesized that topological and non-topological exemplars would be encoded within the HVC and the HC regardless of the task and of the position of the exemplars in the space (i.e., their belonging quadrant). We further predicted that topological stimuli would be better discriminated than non-topological ones (Q3a), in the perception rather than the imagery domain, as well (Q3b).

Regarding the distinction between faces and buildings, according to the distributed view of the object representation within the HVC by Haxby et al. (2001), we predicted that both categories would be largely represented in the areas of the HVC, independently from their selectivity for one category over the other (Q3c).

#### Q3a: Are topological mental images coded better than non-topological ones within the HVC?

This question focused on the mental images, independent of their spatial information at difference with Q2, and aimed at assessing differences in neural dissimilarity (Fig. 5) among categories (i.e., building, city and hour). We performed separate one-tailed *t*-tests (Fig. 9) for each category, resulting in three one-sample *t*-tests for each region. We found significant effects in the left OPA (*t*_14_ = 4.77, *p* = 1.49 × 10^–4^) and the right PPA (*t*_14_ = 3.75, *p* = 1.07 × 10^–3^) for cities, as well as in the right PPA (*t*_14_ = 3.57, *p* = 1.54 × 10^–3^) for hours, and in the bilateral OPA (LH: *t*_28_ = 3.39, *p* = 1.04 × 10^–3^; RH: *t*_28_ = 3.82, *p* = 3.38 × 10^–4^) and the right PPA (*t*_28_ = 3.05, *p* = 2.45 × 10^–3^) for buildings.

Since we hypothesized that the areas within the HVC would discriminate between pairs of buildings better than pairs of exemplars belonging to other categories, we performed a one-tailed two-sample *t*-test in the left OPA (significance level: *p* < 0.05) which did not reveal significant differences. Similarly, we performed a one-way ANOVA in the right PPA (significance level: *p* < 0.05), which did not reveal any preference for one category over the others.

In sum, the right PPA significantly encoded the difference between pairs of cities, hours and buildings to the same extent; the right OPA only discriminated between pairs of buildings; whereas, the left OPA also discriminated between pairs of cities, at the same extent as pairs of buildings.

#### Q3b: Are buildings encoded better during perception than during imagery?

This question focused on spatial encoding of buildings and aimed at assessing differences in neural dissimilarity among domains.

As a first step, we checked whether areas in the HVC and the HC encode the neural dissimilarities (Fig. 7) between pairs of buildings by performing separate one-sample *t*-tests (Fig. 10) in the perception domain, resulting in one comparison for each region. All the areas in the HVC, but not the bilateral HC, discriminated between pairs of perceived buildings (OPA LH: *t*_15_ = 4.21, *p* = 3.76 × 10^–4^; OPA RH: *t*_15_ = 5.89, *p* = 1.49 × 10^–5^; PPA LH: *t*_15_ = 4.84, *p* = 1.08 × 10^–4^; PPA RH: *t*_15_ = 7.88, *p* = 5.21 × 10^–7^; RSC LH: *t*_15_ = 3.35, *p* = 2.21 × 10^–3^; RSC RH: *t*_15_ = 3.44, *p* = 1.82 × 10^–3^).

As a second step, we compared these data to the data resulting from Q3a question relative to the imagery domain (i.e., the dissimilarity between imagined buildings), predicting that perceived buildings would be discriminated better during perception than during imagery. Since the bilateral OPA and the right PPA significantly discriminated both pairs of imagined buildings (Q3a) and of perceived buildings, we performed one-sided two-sample *t*-tests on these areas (*p* < 0.016). Results showed that distances were higher in perception than in imagery in all the areas (OPA LH: *t*_27_ = 2.52, *p* = 7.75 × 10^–3^; OPA RH: *t*_27_ = 4.34, *p* = 4.24 × 10^–5^; PPA RH: *t*_27_ = 5.54, *p* = 8.57 × 10^–7^).

In sum, all the areas in the HVC discriminated between pairs of perceived buildings. Instead, only the bilateral OPA and the right PPA discriminated between imagined buildings, to a lesser extent than during perception.

#### Q3c: Is the amount of similarity between exemplars higher during perception than during imagery, and different across buildings and faces?

We addressed these questions separately for the building category and the face category and for perception and imagery domain (Fig. 4), by performing one-sample *t*-tests (Fig. 11) between exemplars belonging to the same or to different categories and to the same or to different domains, resulting in four analyses for each region. One-sample *t*-tests were significantly higher than zero on the distances between pairs of perceived buildings in the bilateral OFA (LH: *t*_18_ = 4.08, *p* = 3.51 × 10^–4^; RH: *t*_18_ = 7.84, *p* = 1.63 × 10^–7^), the bilateral PPA (LH: *t*_18_ = 6.09, *p* = 4.61 × 10^–6^; RH: *t*_18_ = 7.52, *p* = 2.95 × 10^–7^), the bilateral OPA (LH: *t*_18_ = 3.36, *p* = 1.74 × 10^–3^; RH: *t*_18_ = 3.55, *p* = 1.13 × 10^–3^) and the left FFA (*t*_18_ = 3.83, *p* = 6.08 × 10^–4^). Instead, pairs of imagined buildings were discriminated only by the left OPA (*t*_18_ = 3.19, *p* = 2.55 × 10^–3^), whereas pairs of perceived faces were discriminated by the left OPA (*t*_18_ = 3.88, *p* = 5.47 × 10^–4^) and right PPA (*t*_18_ = 3.14, *p* = 2.83 × 10^–3^). We did not find significant effects in any ROI in imagined faces (*p*_s_ > 0.029).

Then, we performed a paired *t*-test to disentangle whether the left OPA discriminated better perceived than imagined buildings, finding a significant difference (*t*_18_ = 1.94, *p* = 3.41 × 10^–2^).

We also aimed at assessing whether there was a better discrimination of perceived buildings or faces in the left OPA and the right PPA by means of paired *t*-tests (*p* < 0.025), revealing that pairs of perceived buildings were discriminated better than pairs of perceived faces in the right PPA (*t*_18_ = 4.65, *p* = 9.92 × 10^–5^), but not in the left OPA.

In sum, pairs of perceived buildings were discriminated in the bilateral OFA and PPA, as well as in the right OPA and the left FFA; whereas, only the left OPA discriminated between pairs of imagined buildings but to a lesser extent than pairs of perceived buildings. Although no area discriminated between pairs of imagined faces, pairs of perceived faces were discriminated by the left OPA, to the same extent of perceived buildings, and the right PPA, to a lesser extent than perceived buildings.

## Discussion

Here, we aimed to unveil whether the fine-grained spatial and visual information about perceived and imagined exemplars are coded in a distributed or a modular fashion in the HVC, and possible differences between imagery and perception. To this aim, we performed a representational similarity analysis (RSA) by computing a cross-validated distance measure (i.e., the crossnobis estimator) that legitimated us to directly compare categories and domains by means of parametric statistical analysis, at difference with our previous study (Boccia et al. 2019). For easiness of exposition, discussion has been divided into subheadings. First, we provide brief methodological considerations. Then, we discuss the main category of results, namely, those about spatial and visual information.

### Methodological considerations

At difference with previous studies (e.g., Boccia et al. 2019; Lee et al. 2012), here, we attempted at evaluating the neural dissimilarities between objects, buildings and faces using a cross-validated distance measure. Besides providing a measure of the true distance between exemplars, since it employs a multivariate noise normalization, the crossnobis estimator allowed us to test directly our hypotheses by means of parametric statistical analyses.

Our set of preliminary analyses successfully verified the method we implemented here, proving that overall inter-category dissimilarities are encoded better in the perception than in the imagery domain, and that inter-domain dissimilarities are encoded in a distributed fashion, in almost all the areas of the HVC. Results also showed that the HC does not discriminate inter-category dissimilarities during imagery and inter-domain dissimilarities for the face category, proving a less subtle power in encoding neural dissimilarities.

### Fine-grained spatial information in the HVC and the HC

Q2s were mainly aimed at testing spatial representation in the HVC and HC. Q2a revealed that the fine-grained spatial information of non-topological images (i.e., hours on the clock face and cities on the map of Italy) is processed in the left OPA and the right PPA. These regions also code for subtle within quadrant information in the case of the map of Italy. Also, the bilateral OPA and the right PPA discriminated between pairs of buildings belonging to different quadrants, to the same extent as pairs of cities and hours belonging to different quadrants. This result ties well with findings from previous analyses using a different set of regions (i.e., derived from an omnibus contrast) and approaches (i.e., decoding; Boccia et al. 2015), suggesting that spatial information about topological and non-topological mental images is widely coded in the HVC.

Q2b, instead, looked for eventual domain-dependent neural signatures of the spatial information carried by topological stimuli. We found that spatial information about items belonging to the same quadrant was coded in the right OPA and PPA only during perception, to the same extent as those belonging to different quadrants. We may speculate that the right OPA and the right PPA are able to discriminate subtle visual perceptual differences that allow discriminating close buildings in the environment, otherwise indistinguishable. This interpretation is consistent with the present finding that the dissimilarity observed in the bilateral OPA and in the right PPA was higher between perceived buildings than imagined ones. Also, it is consistent with the previous studies finding that these areas are finely tuned to discriminate textural and structural features of the local environment (PPA) and to extract spatial features from visual scenes that can be used to identify the navigational affordances (OPA; Bonner and Epstein 2017).

It is noteworthy that during perception buildings belonging to the same quadrant were discriminated only by the right OPA and PPA, whereas different quadrants were processed within a wide bilateral network of areas, including all the scene-selective regions in the HVC, able to discriminate buildings basing on both visual perceptual differences and their different spatial location. The additional involvement of the left OPA and PPA, as well as the bilateral RSC, ties well with previous findings obtained by a decoding approach (Boccia et al. 2015) and is consistent with the role of the PPA and RSC in spatial navigation (Boccia et al. 2014). Indeed, these regions play different and complementary contributions to human spatial navigation, especially concerning the perceptual processing of salient landmarks (Epstein et al. 2017).

### Fine-grained visual information in the HVC and the HC

At difference with Q2s, which focused on the spatial representation of topological and non-topological contents, Q3s were aimed at testing neural representation of specific exemplars, regardless of their spatial position. Q3a assessed the neural representation of topological and non-topological mental images; results revealed that the right PPA significantly encoded the difference between pairs of cities, hours and buildings to the same extent; the right OPA only discriminated between pairs of buildings; whereas, the left OPA discriminated also pairs of cities, to the same extent of pairs of buildings. This pattern of results expands over the previous ones (Boccia et al. 2015) suggesting that the HVC hosts a mechanism allowing for fine-grained coding of contents in service of mental imagery.

Q3b was mainly set to reveal eventual differences across domains in the neural representation of topological stimuli. This analysis proved that all the scene-selective areas in the HVC discriminated between pairs of perceived buildings; instead, only the bilateral OPA and the right PPA discriminated between imagined buildings, to a lesser extent than during perception. These results deserve two important considerations. On the one hand, finding that scene-selective regions of the HVC are involved in representing dissimilarities between perceived familiar buildings is not surprising, given their specialization (Epstein et al. 2017). On the other hand, finding that the bilateral OPA and the right PPA also code for neural dissimilarities between imagined buildings deserves further consideration, especially about the role of HVC in visual imagery. The activity patterns in the visual cortex have been repeatedly used to decode the individual perceived or imagined exemplar both in the case of objects (Lee et al. 2012) and places (Boccia et al. 2015, 2017; Johnson and Johnson 2014). This result further expands over these previous findings, suggesting that the neural dissimilarities between perceived familiar buildings are also detectable during imagery in the OPA and the PPA.

Previous studies using decoding found that content-dependent regions of the HVC (including the PPA, the FFA, and the OFA) share the representations of the perceptual category (i.e., place or faces) about both preferred and non-preferred categories during perception (Haxby et al. 2001; O'Toole et al. 2005) and imagery (Cichy et al. 2012). As reported in the introduction section, these results tie well with the principle of distributed encoding. In this vein results from Q3c, aimed at comparing neural dissimilarities across categories and domains, provide new evidence for the principle of distributed encoding. Indeed, we found that pairs of perceived buildings were discriminated in the bilateral OFA and PPA, as well as in the right OPA and the left FFA, whereas only the left OPA discriminated between pairs of imagined buildings but to a lesser extent than pairs of perceived buildings. Although no area discriminated between pairs of imagined faces, pairs of perceived faces were discriminated by the left OPA, to the same extent as perceived buildings, and the right PPA, to a lesser extent than perceived buildings.

Also worthy of note is the comparison between the results of the Q3c and those of the Q3a–Q3b questions, relative to the visual information encoded by the scene-selective areas in the HVC during a task that requires to focus on the spatial information represented by the stimuli. Indeed, our results confirm and extend previous studies (Lee et al. 2012) enlightening a gradient of specialization within the scene-selective areas in the HVC, during imagery and perception. One may speculate that the earliest area (i.e., OPA) in the left hemisphere mostly encodes visual perceptual differences, being able to discriminate between buildings in both domains, but better in perception than in imagery, independently from the task; then, the right PPA and OPA are sensitive also to the spatial information, since they successfully encoded the difference between imagined buildings only when the task required to recall their locations (Q3a); a similar selectivity for task demands related to the spatial location of the stimuli was found in the bilateral RSC, but here, at difference with the right PPA and OPA, only in the perception domain the difference between individual buildings was successfully encoded, suggesting that this region mostly relies on the spatial information. This interpretation is also consistent with the results of the Q2s questions, specifically focused on the encoding of the spatial information within the HVC.

### Future directions

Here, we set out with a RSA of previous data to disentangle the way in which spatial and visual information about perceived and imagined exemplars are coded in the HVC and the HC, and possible differences between imagery and perception. Overall we found that spatial information is widely coded in the HVC during perception (i.e., RSC, PPA and OPA) and imagery (OPA and PPA). Also, visual information seems to be coded in both preferred and non-preferred regions of the HVC, supporting a distributed view of encoding. Even if the contribution of different regions of the HVC to scene (Epstein et al. 2017) and face perception (Liu et al. 2010) has been widely investigated, little is known about the specific contribution of each region to mental imagery. Indeed, recent models (see Pearson (2019) for a recent review) have mainly focused on the crucial role of early visual area, as V1 and V2, in visual mental imagery. In particular, it has been demonstrated that the content of mental imagery can be decoded from these early visual areas (Naselaris et al. 2015; Koenig-Robert and Pearson 2019) and that the excitability of early visual cortex predicts imagery strength (Keogh et al. 2020), thus supporting a key role of this cortex in defining precise features of visual imagery (Pearson 2020). Thus, future studies should test the specific contribution of different regions, such as the early visual areas, and that of the underlying neural representation, to mental imagery.

Left hemisphere lateralization of some results concerning mental imagery is broadly consistent with evidence from patients with extensive left temporal damage and impaired visual mental imagery. Indeed, in the presence of intact primary visual area, lesion in the left temporal lobe has been associated with pure visual mental imagery deficits (Moro et al. 2008). Future studies should expand over the contribution of the left hemisphere to visual mental imagery, especially by integrating causal evidence from brain damaged patients.

## Supplementary Information

Below is the link to the electronic supplementary material.

Supplementary file1 (DOCX 10416 KB)

## Data Availability

Data are available upon request to the corresponding author in compliance with the institutional ethics approval.

## Code availability

Scripts used for running experiments and data analyses are available upon request to the corresponding author.
