# Supplementary material for: Neural representations underlying mental imagery as unveiled by representation similarity analysis
Source: Brain Struct Funct. 2021 Apr 5;226(5):1511–31. doi: 10.1007/s00429-021-02266-z (PMC8096739; doi:10.1007/s00429-021-02266-z)
Supplement: Supplementary file 1 — Supplementary file1 (DOCX 10416 KB) [file 429_2021_2266_MOESM1_ESM.docx]

**Figure S1** - *Design and timeline of Experiment 1*. **A**. Stimuli from the map of the campus, map of Italy and clock. **B**. Experimental timeline: each of the five fMRI scans consisted of 120 experimental trials (imagery trials), plus 5 null trials and 9 question trials.

**Figure S2** - *Design and timeline of Experiment 2*. **A**. Examples of stimuli to be watched or imagined. **B**. Experimental timeline: each of the three scans consisted of 48 perceptual and 48 imagery experimental trials, plus 12 null trials and 6 question trials.

**Figure S3** - *Design and timeline of Experiment 3*. **A**. Examples of stimuli to be watched or imagined. **B**. Experimental timeline: each of the five scans consisted of 60 perceptual (photos) and 60 imagery (labels) trials (half were faces), plus 5 null trials and 9 question trials. Written informed consent for publication of identifying images was obtained from the individual represented in the figure.

**Figure S4** - Overlap between *scene-selective* (A) and *face-selective* (B) regions derived from our localizer analyses (white line) and Z transformed posterior probability for the terms “navigation” and “face” (hot maps in panels A and B, respectively). Statistical inference maps were obtained using neurosynth (Yarkoni et al., 2011) and show z-scores for the uniformity test maps; thus, they display the degree to which each voxel is consistently activated in studies that use a given term.

**Reference**

Yarkoni, T., Poldrack, R.A., Nichols, T.E., Van Essen, D.C. & Wager T. (2011) Large-scale automated synthesis of human functional neuroimaging data. Nature Methods 8, 665–670
